# A possible case of primary renal lymphoma: a case report

**DOI:** 10.4076/1757-1626-2-6233

**Published:** 2009-07-29

**Authors:** Germar-Michael Pinggera, Reinhard Peschel, Alexander Buttazzoni, Michael Mitterberger, Aigner Friedrich, Leo Pallwein

**Affiliations:** Department of Urology and Radiology, Medical University InnsbruckAnichstrasse 35, Innsbruck, 6020Austria

## Abstract

**Introduction:**

The entity primary renal lymphoma is controversial and rare.

**Case presentation:**

We report a case in a 60-year-old man. Computed tomography revealed a large, homogeneous, retroperitoneal mass with 14.8 × 11.5 cm size arising from the right kidney. An ultrasound guided percutaneous biopsy was performed and the tumour was diagnosed histopathological as non-Hodgkin lymphoma. The patient was treated by systemic chemotherapy and thereafter a nephrectomy was performed.

**Conclusion:**

Primary renal lymphoma is a controversial and infrequent disease. However, there is growing evidence that it does exist.

## Introduction

Renal involvement is frequently seen in patients with lymphoma. However, the entity primary renal lymphoma (PRL) is controversial and rare. The term PRL is applied when the disease is localized to the kidney without any sign of other organ involvement or in whom renal involvement is the presenting manifestation [1]. We report a questionable case of a PRL and discuss this rare entity.

## Case presentation

A 60-year-old Caucasian male from Austria presented with dyspnoea, intermitted claudication and fatigue. In the last 2 months he noticed an unvoluntary weight loss of 15 kg. Physical examination showed an indolent resistance on the right flank. Blood sample in a peripheral vein showed results within normal ranges (haemoglobin 13.1 g/dl, white blood cell count 6.7 G/l; polymorphonuclear cells 63.5%, lymphocytes 13.5%, monocytes 9.0%, eosinophils 13.0%, basophils 0.5%, platelet count 158 G/l; serum creatinine 1.16 mg/dl), whereas urinary analysis showed microhaematuria and proteinuria.

An ultrasound (US) examination (Acuson Sequoia, California, USA) of the abdomen and pelvis showed a large and hypoechoic retroperitoneal mass surrounding the right kidney with extension into the right renal hilum and no evidence of urinary obstruction. The contralateral kidney appeared to be normal.

**Figure 1.**
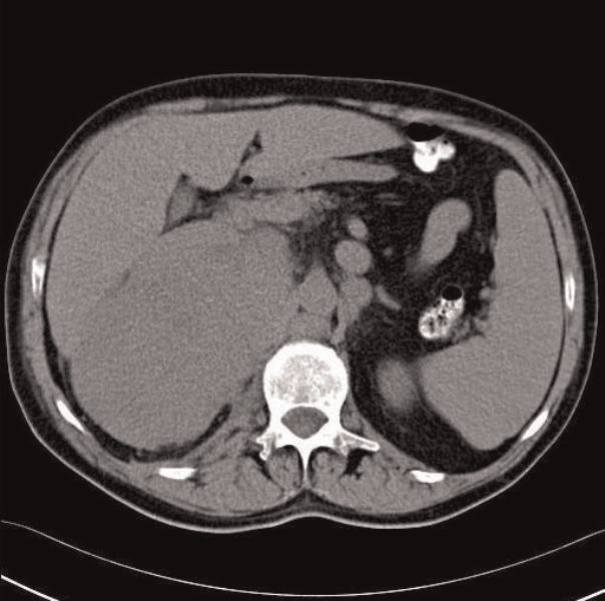
Abdominal CT scan (native) image showing hypodense renal mass involving the right kidney with perinephric extension.

**Figure 2.**
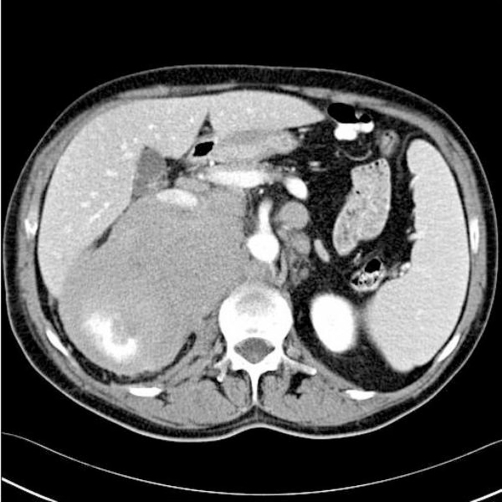
Abdominal CT (contrast enhanced) scan image showing hypodense renal mass involving the right kidney with perinephric extension.

A 4-row helical CT examination (4 Volume Zoom, Siemens, Erlangen, Germany) of the whole body with a standard examination protocol showed a retroperitoneal mass (with 14.8 × 11.5 cm size) from the right kidney and infiltrating Gerota’s fascia (see Fig 1 & 2). Lymphadenopathy was detected in the lower part of mediastinum and in the retroperitoneal space with borderline size values. Beside infitrative destruction of the flanking right rip no further infiltration of other organs or metastasis was found. The volume of liver and spleen was within normal range.

Scintigraphically, a radioisotope Tc-99m bone scan showed no suspicious lesions.

US guided percutaneous biopsy of the retroperitoneal mass was performed under local anaesthesia. Immunohistochemical stains were positive for bcl-2, bcl-6, CD10, CD20 and negative for CD 5, CD23, Cyklin D1. The histological diagnosis was a grad 2 low proliferating follicular non-Hodgkin lymphoma (NHL). Chemotherapy was started according to the CHOP scheme. The tumor responded well to the chemotherapy and about 70% of regression was achieved after six courses of chemotherapy. Thereafter a nephrectomy with complete lymph node dissection and dissection of the retroperitoneal mass has been performed. Final diagnosis was a primary renal non-Hodgkin lymphoma (NHL).

## Discussion

PRL is rare and its entity controversial [2]. In autopsy series, estimates of renal involvement in patients with known lymphoma range from 30% to 60% [3]. Kidneys can be the primary site of disease or a site of disseminated extranodal involvement. Common extranodal sites include the kidneys, bone marrow, liver, and gastrointestinal tract. However renal involvement is detected in only 3%-8% of all patients undergoing routine computed tomography (CT) staging for lymphoma. This discrepancy reflects the fact that patients with presumed lymphomatous renal involvement rarely undergo nephrectomy or biopsy, and disease involvement is often poorly documented [4,5]. Renal involvement with lymphoma occurs much more commonly with non-Hodgkin disease. The majority of patients have intermediate or high-grade lymphomas, most of them of B-cell origin. Involvement usually occurs late in the course of the disease and is clinically often silent. Occasionally, patients present with nonspecific signs and symptoms including flank pain, weight loss, hematuria, or a palpable mass. The evaluation of renal lymphoma is important and includes differentiation from other renal malignancies, timely provision of a pathologic diagnosis and preservation of renal parenchyma and function [6].

Several imaging options exist for evaluation of renal involvement including ultrasonography, intravenous urography, CT, nuclear medicine and magnetic resonance imaging. US image demonstrate lymphoma with a characteristic hypoechoic appearance, a finding that reflects homogeneity. However, CT remains the most sensitive, efficient, and comprehensive examination for evaluation of the kidneys and is the imaging modality of choice in patients with suspected renal masses including renal lymphoma and also for definition of extrarenal extent of disease [6]. The typical CT patterns in renal lymphoma include following [1-7]: multiple renal masses (with up to 60 percent of the cases), solitary masses (rarest with below 6% of cases), renal invasion from contiguous retroperitoneal disease (seen in approximately 25-30% of patients), perirenal disease, or diffuse renal infiltration (almost always bilateral and is seen in approximately 20% of patients) [7]. Atypical findings include spontaneous hemorrhage, necrosis, heterogeneous attenuation, cystic transformation, and calcification. CT findings in renal lymphoma are often nonspecific and may be seen with a variety of benign and malignant conditions [7]. Solid renal masses including renal cell carcinoma and metastases are the most commonly encountered entities that mimic renal lymphoma at CT [8]. Renal cell carcinoma tends to have a more heterogeneous appearance than renal lymphoma. Interruption of the enhancing cortical rim suggests an underlying renal mass as the cause of bleeding. These findings are typical in renal cell carcinoma but very unusual in lymphoma. Metastases from primary tumors such as lung cancer, breast cancer, or synchronous renal cell cancer often manifest as bilateral masses that are indistinguishable from multifocal lymphoma. In such cases, a history of primary malignancy is essential for accurate diagnosis. Nevertheless, many lesions have overlapping CT features and require biopsy for definitive diagnosis [6]. An infiltrative growth pattern may be seen with tumors such as transitional cell carcinoma or with inflammatory processes such as acute pyelonephritis or xanthogranulomatous pyelonephritis. The presence of multisystemic disease should always raise suspicion for lymphoma [9].

Standard management of a renal mass is nephrectomy. PRL is an rare exception in which patient should be treated first with chemotherapy. An early diagnosis and management can help to improve outcome in these patients.

## Abbreviations

NHL: Non-Hodgkin lymphoma
PRL: Primary renal lymphoma
